# Role of H4K16 acetylation in 53BP1 recruitment to double-strand break sites in in vitro aged cells

**DOI:** 10.1007/s10522-022-09979-6

**Published:** 2022-07-18

**Authors:** Lourdes González-Bermúdez, Anna Genescà, Mariona Terradas, Marta Martín

**Affiliations:** 1 Departament de Biologia Cel·lular, Fisiologia i Immunologia, Facultat de Biociències, Universitat Autònoma de Barcelona, Bellaterra, Spain; 2 Hereditary Cancer Program, Catalan Institute of Oncology, IDIBELL, Hospitalet de Llobregat, Spain

**Keywords:** H4K16 acetylation, 53BP1, In vitro aging, Double strand break repair, DNA damage

## Abstract

Increased frequency of DNA double strand breaks (DSBs) with aging suggests an age-associated decline in DSB repair efficiency, which is also influenced by the epigenetic landscape. H4 acetylation at lysine 16 (H4K16Ac) has been related to DSB repair since deacetylation of this mark is required for efficient 53BP1 recruitment to DSBs. Although age-associated changes in H4K16Ac levels have been studied, their contribution to age-related DSB accumulation remains unknown. In vitro aged Human Dermal Fibroblasts (HDFs) display lower levels of H4K16A that correlate with reduced recruitment of 53BP1 to basal DSBs. Following DNA damage induction, early passage (EP) cells suffered from a transient H4K16 deacetylation that allowed proper 53BP1 recruitment to DSBs. In contrast, to reach this specific and optimum level, aged cells responded by increasing their overall lower H4K16Ac levels. Induced hyperacetylation of late passage (LP) cells using trichostatin A increased H4K16Ac levels but did not ameliorate 53BP1 recruitment. Instead, deacetylation induced by MOF silencing reduced H4K16Ac levels and compromised 53BP1 recruitment in both EP and LP cells. Age-associated decrease of H4K16Ac levels contributes to the repair defect displayed by in vitro aged cells. H4K16Ac responds to DNA damage in order to reach a specific, optimum level that allows proper 53BP1 recruitment. This response may be compromised with age, as LP cells depart from lower H4K16Ac levels. Variations in H4K16Ac following the activation of the DNA damage response and aging point at this histone mark as a key mediator between DNA repair and age-associated chromatin alterations.

**Supplementary Information:**

The online version contains supplementary material available at 10.1007/s10522-022-09979-6.

## Introduction

To efficiently maintain genome integrity and prevent the acquisition and transmission of mutations and genome aberrations, cells have evolved a complex signaling network termed as DNA damage response (DDR) (Jackson and Bartek 2009). Proper function of the DDR is crucial for rapid detection and repair of various types of DNA damage. Double strand breaks (DSBs) represent one of the most deleterious types of DNA lesions because they can lead to DNA fragment loss and to the formation of chromosomal rearrangements and mutations (Polo and Jackson 2011). Accumulation of DSBs has been reported in human cells aged in vitro, such as fibroblasts (Sedelnikova et al. 2008) and human mammary epithelial cells (HMECs) (Hernández et al. 2013). Persistent DSBs have also been found in different human cell types from aged donors such as hematopoietic stem cells (Rübe et al. 2011), lymphocytes (Sharma et al. 2010), fibroblasts (Kalfalah et al. 2015) and HMECs (Anglada et al. 2019). In eukaryotes, DSBs are repaired by two major DDR pathways: the homologous recombination (HR) pathway, which operates during S and G2 phases of the cell cycle, and the nonhomologous end-joining (NHEJ) pathway, which operates throughout the cell cycle (Huertas 2010). An age-associated decline in the efficiency of NHEJ repair has been reported in mice (Vaidya et al. 2014), rats (Vyjayanti et al. 2006), human senescent cells (Seluanov et al. 2004) and human cells from aged donors (Anglada et al. 2020). These results strongly suggest a link between aging and DSB repair efficiency.

Chromatin conformation has emerged as an essential factor in the regulation of both HR and NHEJ. Histone modifications such as acetylation, methylation, phosphorylation and ubiquitylation can alter the chromatin state and, hence, influence the performance of the DDR. Faced with a DSB, the ataxia-telangiectasia mutated (ATM) kinase phosphorylates the histone H2AX in chromatin regions flanking nascent DSBs (Misteli and Soutoglou 2009). The phosphorylated form of H2AX, γH2AX, is a widely accepted marker of DSBs and is essential for the recruitment of DNA damage signaling proteins to the break site, such as mediator of DNA damage checkpoint protein 1 (MDC1) (Misteli and Soutoglou 2009). Subsequent H2A ubiquitylation by the ubiquitin E3 ligase RNF8 at DSB sites promotes the recruitment of DNA repair proteins, such as p53 binding protein 1 (53BP1) and breast cancer type 1 susceptibility protein (BRCA1), to DSBs (Mailand et al. 2007; Doil et al. 2009). The focal accumulation of 53BP1 relies on the specific binding of the 53BP1 tandem Tudor domain to the dimethylated lysine 20 of histone H4 (H4K20me2) (Sanders et al. 2004; Botuyan et al. 2006; Yang et al. 2008). On the contrary, lysine 16 acetylation of the same histone H4 (H4K16Ac) significantly reduces 53BP1 binding to H4K20me2 by disrupting a salt bridge that is necessary for the association of the Tudor domain of the protein with the H4 tail (Tang et al. 2013; Hsiao et al. 2013).

One of the key roles of H4K16Ac is its capacity to inhibit chromatin compaction and generate open and accessible chromatin, which explains its variable levels throughout the cell cycle (Shogren-Knaak et al. 2006). H4K16Ac peaks during S phase, when DNA replication requires an open chromatin state, and decreases at G2/M, reaching its lowest levels during mitosis coinciding with the metaphase chromosome formation (Vaquero et al. 2006). Regarding the relationship between the acetylation state of H4K16 and DSB repair, different studies have revealed complex H4K16Ac dynamics after DNA damage induction. For instance, a global H4K16 acetylation has been reported after DSB induction by ionizing radiation (Li et al. 2010; Sharma et al. 2010). However, another work has described a transient H4K16 deacetylation after DSB induction that returned to basal levels 2 h post-irradiation and that was mediated by the histone deacetylases HDAC1 and HDAC2. The authors proposed that this transient H4K16 deacetylation might contribute to recruitment of 53BP1 to DSBs, favoring their repair through the NHEJ pathway (Miller et al. 2010). Overall, these results highlight the complexity of H4K16Ac regulation in response to DNA damage.

The relevance of H4K16Ac is not restricted to the regulation of DNA damage repair, as changes in H4K16Ac levels have also been related to aging. Cellular senescence is associated with global deacetylation of H4K16, which can contribute to DNA compaction in senescent cells, leading to profound modulating effects on DNA transcription (Contrepois et al. 2012). H4K16Ac reduction has also been observed in a mouse model of premature aging (Krishnan et al. 2011). Additionally, genome-wide analysis of H4K16Ac patterns in human brain tissue has demonstrated a redistribution of this mark during normal aging (Nativio et al. 2018).

The fact that aging has been linked to accumulation of DSBs, impaired recruitment of 53BP1 to DSBs (Hernández et al. 2013; Anglada et al. 2020) and changes in the acetylation state of H4K16 (Krishnan et al. 2011) prompted us to evaluate the contribution of H4K16Ac to the age-related increase of DSBs. Here, we describe that age-associated DNA damage repair deficiency is accompanied by impaired H4K16 acetylation in human dermal fibroblasts (HDFs) during in vitro aging. Furthermore, we observe that H4K16Ac levels reach a specific and stable H4K16 acetylation level after DNA damage induction that is required for proper 53BP1 recruitment. In order to expand the knowledge concerning H4K16Ac dynamics, this study provides further insights into the involvement of H4K16Ac in regulation of the DDR during cellular aging.

## Methods

### Cell culture and treatments

HDFs were obtained from Cell Applications (Cat.#: 106-05n). HDFs were cultured in DMEM (Biowest) supplemented with 10% fetal bovine serum (FBS), 1% GlutaMAX and 1% penicillin–streptomycin (Thermo Fisher Scientific Inc.). All cell types were incubated under the conditions of 37 °C and 5% CO2 atmosphere. When indicated, cells were treated with the following chemicals: 10 µg/ml Bleocine (Millipore) for 1 h, 1 µM trichostatin A (TSA; Sig-ma‐Aldrich) for 12 h and 5 mM nicotinamide (Sigma‐Aldrich).

### Cell cycle analysis by flow cytometry

Cells were fixed in ethanol and stored at − 20 °C. Cells were stained with propidium iodide staining solution (0.1% Triton-X-100 in phosphate buffered saline (PBS) solution, 0.2 mg/ml DNase-free RNase A and 0.02 mg/ml propidium iodide). After 30 min of incubation at room temperature (RT), fluorescence intensity was measured using a FACSCalibur cytometer (Becton Dickinson) and analyzed using FlowJo software.

### Fluorescent immunodetection

Mouse monoclonal γH2AX (1:1000; Millipore,), rabbit polyclonal 53BP1 (1:2000; Abcam) and rabbit polyclonal H4K16Ac (1:200; Active Motif) were used for γH2AX, 53BP1 and H4K16Ac detection. Cells were washed with 1 × PBS, fixed with 4% paraformaldehyde (PFA) at RT (room temperature) and permeabilized in 0.5% Triton X-100. To detect H4K16Ac, antigen retrieval treatment was performed by incubating the cells with a target retrieval solution (Agilent) for 1 h at 65 °C. Cells were blocked for 1 h with 0.5% BSA-0.15% glycine in PBS, incubated overnight with primary antibodies at 4 °C and incubated for 1 h at RT with secondary anti-mouse Cy3-conjugated (1:1000; Jackson IR) and anti-rabbit Alexa 488-conjugated (1:1000; Thermo Fisher Scientific Inc.) antibodies. If a third antibody was detected (H4K16Ac), secondary antibodies from the previous two immunodetections were reapplied and incubated. The secondary antibody for H4K16Ac (anti-rabbit Alexa 532-conjugated; 1:1000; Thermo Fisher Scientific Inc.) was then incubated for 30 min at RT. Slides were rinsed in distilled water, alcoholically dehydrated and mounted on microscope slides using Vectashield mounting medium for fluorescence (Vector Laboratories) supplemented with 0.25 μg/ml 4,6-diamino-2-phenylindole (DAPI). Slides were analyzed with an epifluorescence microscope (BX61; Olympus) equipped with a Plan-Apochromat 100 × NA 1.4 oil objective and the Cytovision software (Applied Imaging). Images were analyzed using ImageJ software (Schneider et al. 2012). To compare fluorescence intensity levels from different immunostainings, identical image acquisition settings were applied to all analyzed slides. The corrected total cell fluorescence was calculated following the protocol used by Gavet and Pines (Gavet et al. 2010).

### Protein extraction and western blotting

For total protein extraction, cells were washed with cold PBS and lysed in SDS lysis buffer. The lysates were sonicated at low intensity and centrifuged at 12,000 rcf for 2 min at 4 °C. The supernatant containing total cell extract was quantified using the BCA protein assay (Thermo Fisher Scientific Inc.). Equal amounts of protein were run in SDS-PAGE on 10% Bis–Tris NuPAGE gels, transferred to PVDF membranes (Thermo Fisher Scientific Inc.), blocked in 5% BSA and incubated with primary and secondary antibodies diluted in 3% BSA-0.1% Tween-20 in PBS. The primary antibodies used for immunoblotting were: mouse monoclonal γH2AX (1:1000; Millipore), rabbit polyclonal H4K16Ac (1:1000; Active Motif), rabbit monoclonal MOF (1:1000; Abcam) and rabbit polyclonal H3 (1:15,000; Abcam). Primary antibody incubation was performed overnight at 4 ºC. For H3 detection, membranes were incubated with 1:15,000 primary antibody for 1 h at RT. Anti-mouse and anti-rabbit secondary antibodies conjugated with horseradish peroxidase (Millipore) were used at a dilution of 1:5000 and incubated for 1 h at RT. Peroxidase activity was detected with Immobilion Western Chemiluminescent HRP Substrate (Millipore). Images were acquired with the ChemiDoc XRS + system using Quantity One software (Bio-Rad).

### RNA collection and RT-qPCR

Total RNA was isolated from cells using TRIzol reagent (Thermo Fisher Scientific Inc.,). To separate RNA from DNA and proteins, 1:5 (v/v) of chloroform was added and the suspension was centrifuged at 12,000 rpm for 15 min. Next, 200 µL of the resulting aqueous phase was mixed with lysis solution from the Maxwell RSC simplyRNA kit (Promega). RNA was extracted with the Maxwell system following the manufacturer’s instructions (Promega). RNA concentration was evaluated with a NanoDrop 2000 spectrometer (Thermo Fisher Scientific Inc.). Single-stranded cDNA was synthesized from 1 µg of total RNA using the iScript cDNA synthesis kit (Bio-Rad) following the manufacturer's instructions. RT-qPCR was performed using universal SYBR Green Supermix (Bio-Rad) in a CFX96 thermal cycler with Bio-Rad CFX Manager software. The amplification program was initiated at 95 °C for 3 min, followed by 40 cycles of 10 s at 95 °C and 30 s at 60 °C. Primer sequences were designed using Primer3 online software (Koressaar et al. 2007)] and are detailed in Table S1. Fold change value was obtained using the 2–ΔΔCt method.

### Sh-RNA expressing plasmids

Two pLKO TRC cloning vector plasmids, containing two different shRNAs to silence MOF, were kindly provided by Alex Vaquero (Josep Carreras Leukaemia Research Institute, Barcelona, Spain) together with a shRNA scramble. Specifically, the shRNA sequences were: 5'-GCAAGATCACTCGCAACCAAA-3' for shMOF1 and 5'- CGAAATTGATGCCTGGTATTT-3' for shMOF2. The plasmids were transformed into E. coli DH5α competent cells, amplified and purified following the instructions of the Library Efficiency DH5α Competent Cells kit (Thermo Fisher Scientific Inc.).

### Lentivirus generation and infection

293 T packaging cells were grown in T75 culture flasks using DMEM (Biowest) containing 10% FBS and 1% penicillin–streptomycin (Thermo Fisher Scientific Inc.). Transfection of 293 T cells was carried out using the calcium phosphate precipitation method. Then, a mixture of the lentiviral transfer vector DNA, together with psPAX2 packaging and pMD2.G envelope plasmid DNA, was prepared at a ratio of 4:3:1, respectively. The final solution was incubated at RT for 15 min and subsequently added onto the cells. Twenty-four hours after transfection, the medium was replaced with DMEM (Biowest) supplemented with 20% FBS and, 48 h after transfection, the medium was harvested and cleared through a 0.2 µm filter. Then, HDFs were infected with the collected medium for 48 h.

### Statistical analysis

Statistical procedures and graph plotting were performed in GraphPad Prism 6.01. Throughout this work, we applied various statistical tests including the Student’s t-test, Mann–Whitney test and Kruskal–Wallis test with Dunn’s correction for multiple comparisons. We have also applied one-way analysis of variance (ANOVA) with Tukey test for multiple comparison. Differences were considered statistically significant when p < 0.05.

## Results

### Deficient 53BP1 recruitment to DSBs during in vitro aging correlates with diminished H4K16 acetylation

**Fig. 1:**
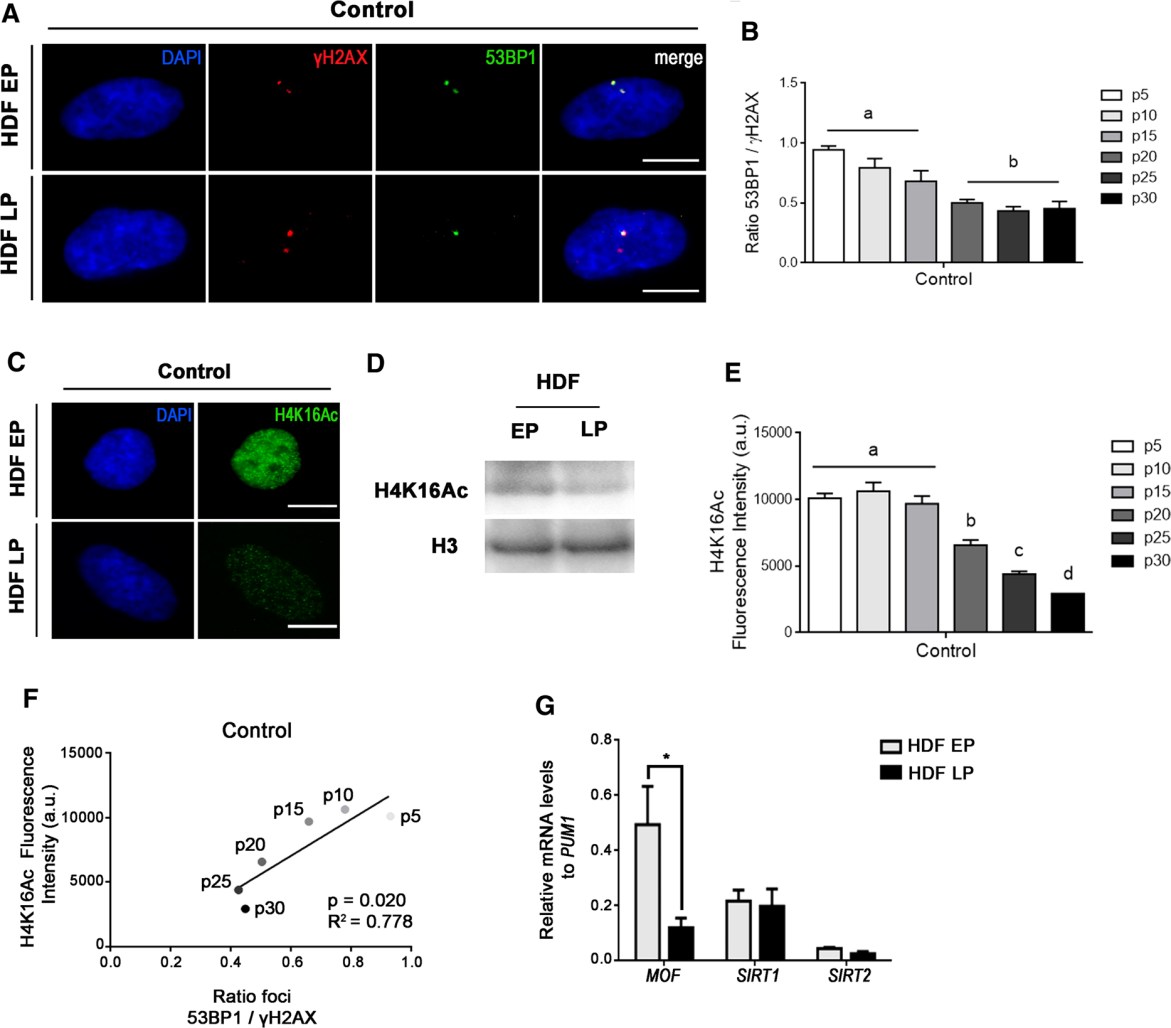
Age-related deficiency in 53BP1 recruitment is associated with a decrease in H4K16 acetylation. **A** Immunostaining of γH2AX (Cy3, red) and 53BP1 (A488, green) foci in EP and LP HDFs under control conditions. The merged panels show γH2AX foci colocalizing with 53BP1 foci. **B** Mean of the ratios of 53BP1 and γH2AX foci colocalization in control HDFs at various culture passages. Error bars indicate SEM and statistically significant differences are indicated by different letters (One-way ANOVA with Tukey test for multiple comparison; p < 0.05). **C** Immunostaining of H4K16Ac (A488, green) in EP and LP HDFs in control conditions. **D** Western blot for H4K16Ac in EP and LP HDFs. Total H3 was used as a loading control. **E** Mean of corrected total cell fluorescence intensity of H4K16Ac in control HDFs at various culture passages. Error bars indicate SEM and statistically significant differences are indicated by different letters (Kruskal–Wallis test with Dunn’s correction; p < 0.05). **F** Correlation between H4K16Ac fluorescence intensity and 53BP1/γH2AX colocalization ratio of HDFs at different culture passages. Correlation coefficient (R2) and p-value were obtained after a linear regression analysis. **G** Relative expression level of MOF, SIRT1 and SIRT2 mRNAs in EP and LP HDFs. Bar graph shows mean and SEM. p-values indicate the Student’s t-test significance levels; *p < 0.05. All the experiments were done in triplicates with 100–150 cells per experiment. DAPI (blue) was used to stain cell nuclei. Scale bar = 10 µm. a.u.: arbitrary units; EP: early passage (culture passage < 10); LP: late passage (culture passage > 20)

An age-associated repair deficiency is widely acknowledged as cells from aged donors present a higher frequency of basal (non-induced) foci of γH2AX, a surrogate marker of DSBs (Anglada et al. 2019). Additionally, defective 53BP1 recruitment to DSBs has been described in epithelial cells from aged donors (Anglada et al. 2020). In order to investigate whether this deficiency also occurs in in vitro aged cells, we analyzed γH2AX foci formation in nuclei of early-passage (EP; culture passage < 15) and late-passage (LP; culture passage > 20) HDFs. Our results show that the frequency of γH2AX foci increased as cell passage increases (Fig. 1A, Table S2). For each γH2AX foci, we then assessed colocalization with 53BP1 foci. Next, we calculated the ratio between the frequencies of 53BP1 and γH2AX foci (Table S2). In HDFs, the 53BP1-γH2AX colocalization ratio gradually decreased with the number of passages in culture, from 0.93 in the earliest passage (p5) to 0.45 in the last in vitro aged passage (p30) (Fig. 1B). Thus, our results demonstrate that the previously reported age-associated defective 53BP1 recruitment to DSBs also takes place during in vitro aging of human fibroblasts and might contribute to the observed accumulation of unrepaired DSBs in these cells.

High levels of H4K16Ac have been described to facilitate chromatin accessibility, and they also play a prominent role in the regulation of 53BP1 foci formation. Therefore, we next investigated the contribution of H4K16Ac status to the age-associated impairment in 53BP1 recruitment. H4K16Ac levels in the whole nucleus of EP and LP HDFs were assessed by immunofluorescence. We observed that H4K16Ac gradually decreases with the number of passages in culture and is significantly reduced from EP (p5, p10, p15) to later passages (p20, p25 and p30) (Fig. 1C and E). Therefore, as cells accumulate passages, the decrease of H4K16Ac levels strongly correlates with a reduction in 53BP1 recruitment to basal γH2AX-labelled DSBs (p-value = 0.020; R2 = 0.778) (Fig. 1F). Reduction of H4K16Ac levels during in vitro aging was also observed by Western blot (Fig. 1D), confirming the immunofluorescence results. Since it has been described that the H4K16 acetylation state is cell cycle-dependent (Shogren-Knaak et al. 2006), we checked the proportions of EP and LP HDFs in each phase of the cell cycle and verified that EP and LP HDFs showed no differences in cell cycle phase distribution (Figure S1).

To gain insight into the molecular mechanism underlying the H4K16Ac reduction with in vitro aging, we analyzed the expression of MOF, the principal H4K16 histone acetyltransferase (Taipale et al. 2005; Smith et al. 2005), and the main H4K16 deacetylases, SIRT1 and SIRT2 (Vaquero et al. 2007), by RT-qPCR (Fig. 1G). We also examined the expression of TIP60, another acetyltransferase of H4K16 (Tang et al. 2013), and the expression of general histone deacetylases HDAC1 and HDAC2 (Miller et al. 2010) (Figure S2). SIRT1 and SIRT2 mRNA levels remained constant during in vitro aging, while MOF expression significantly decreased with time in culture (Fig. 1G). Regarding the expression of TIP60, HDAC1 and HDAC2, no differences were detected (Figure S2). However, it should be taken into consideration that these results show mRNA expression and that they are not necessarily reflecting the levels of protein codified by these genes.

Taken together, these results identify deficient 53BP1 recruitment to DSBs and reduction of H4K16 acetylation as common markers of in vitro aged fibroblasts. Moreover, this evidence seems to point to changes in the expression level of MOF as a mechanism involved in H4K16Ac reductions during in vitro aging.

### Differential H4K16 acetylation dynamics in early and late passage HDFs after DSBs induction

**Fig. 2:**
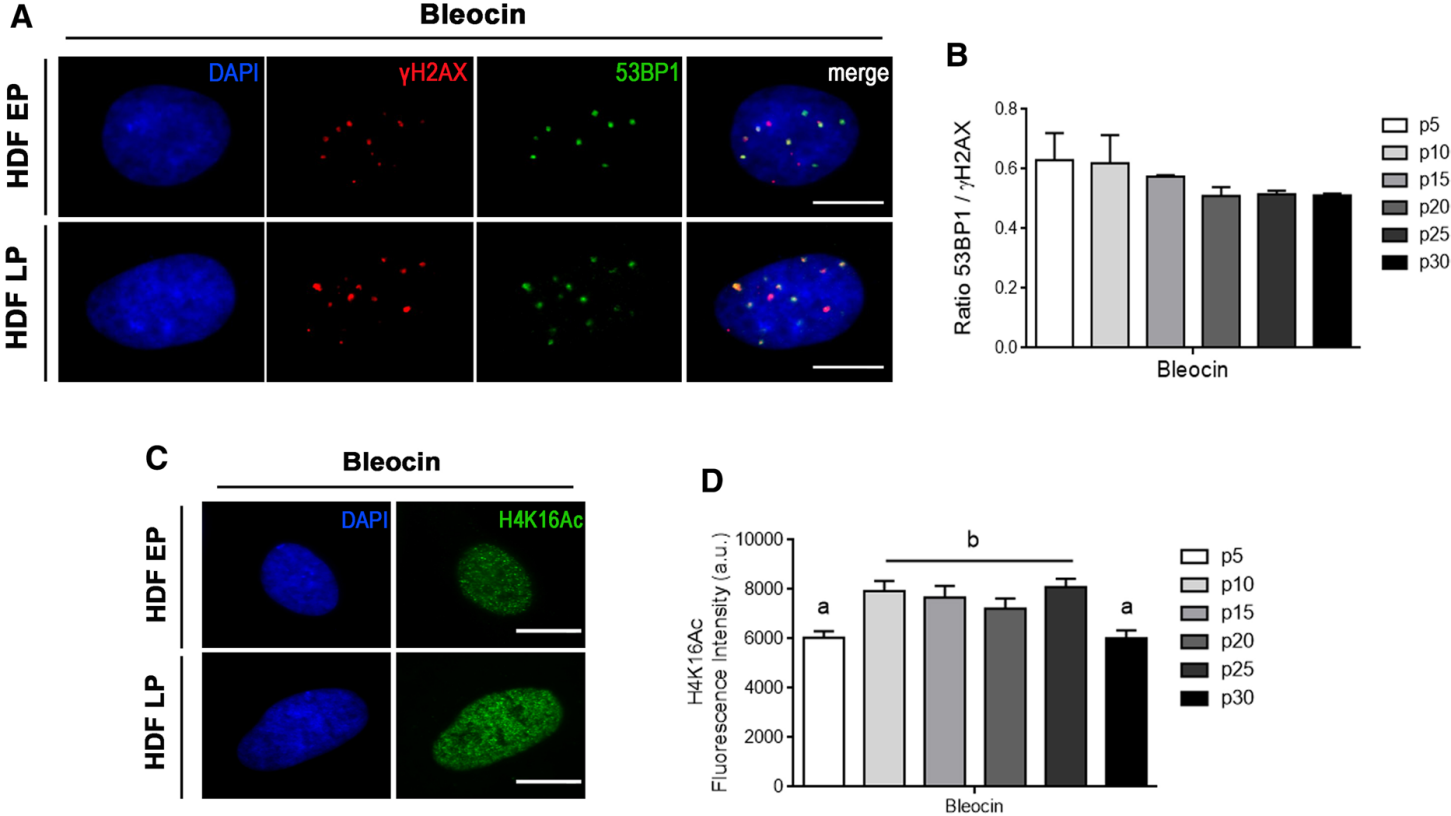
53BP1 recruitment after DSB induction is related to H4K16 acetylation level. **A** Double immunofluorescence detection of γH2AX (Cy3, red) and 53BP1 (A488, green) foci in EP (culture passage < 10) and LP HDFs (culture passage > 20) after Bleocin treatment. The merged panels show γH2AX foci colocalizing with 53BP1 foci. **B** Mean of the ratios of 53BP1 and γH2AX foci colocalization in Bleocin-treated HDFs at various culture passages. **C** Immunostaining of cell nuclei with H4K16Ac (A488, green) in EP and LP HDFs after Bleocin treatment. **D** Mean of corrected total cell fluorescence intensity of H4K16Ac in Bleocin-treated HDFs at different culture passages. Error bars indicate SEM and statistically significant differences are indicated by different letters (Kruskal–Wallis test with Dunn’s correction; p < 0.05). All the experiments were done in triplicates with 100–150 cells per experiment

Because in vitro aged HDFs showed a higher frequency of basal (non-induced) γH2AX foci devoid of 53BP1 than EP cells did, we next used the radiomimetic agent Bleocin to induce DSBs and assessed the subsequent recruitment of DNA repair proteins in EP and LP HDFs. γH2AX and 53BP1 foci formation were assessed 1 h after treatment in various culture passages via double γH2AX and 53BP1 immunodetection. As expected, Bleocin treatment resulted in increased γH2AX foci frequency, both in EP and LP cells (Fig. 2A and Table S3). Nonetheless, the increase was higher in LP HDFs (p20 to p30), suggesting that either older cells are more sensitive to Bleocin or that, in LP HDFs, Bleocin-induced DSBs are added to unrepaired basal DSBs already present. The colocalization ratio of 53BP1 and γH2AX foci, 1 h after Bleocin treatment, was ~ 0.72 in p5 HDFs (Fig. 2B), which was a lower value than that obtained in untreated p5 HDFs (~ 0.93) (Fig. 1B). We presume that the lower colocalization ratio of 53BP1 foci with γH2AX foci in EP cells treated with Bleocin results from the constant presence of the drug in the culture during the experiment. Some DSBs are induced at the beginning of the treatment and, thus, have been able to recruit 53BP1. However, other DSBs were presumably induced just minutes before finishing the treatment and, thus, have not yet been able to recruit 53BP1. In this regard, in a previous work from our group, we described 53BP1 recruitment kinetics to DSBs. Fifteen minutes after DSB induction, 53BP1 recruitment was at half capacity and then steadily increased until reaching a plateau 60 min after DSB induction (Anglada et al. 2020). Because passage 5 and passage 10 HDFs are repair competent EP cells with no repair defects and their colocalization ratio is the same, we assume that ~ 0.7 is the highest colocalization ratio that can be achieved after continuous exposure of HDFs to Bleocin during 1 h. Interestingly, LP cells showed a higher 53BP1-γH2AX colocalization ratio after Bleocin treatment than in control conditions (Figs. 1B and 2B). These results suggest that some event related with the triggering of the DDR is favoring 53BP1 recruitment to DSBs in in vitro aged cells.

Global H4K16 deacetylation has been reported in response to DSB induction (Li et al. 2010), which may favor 53BP1 recruitment within minutes following DNA damage induction. Other works have described a biphasic pattern in H4K16Ac, with an initial wave of deacetylation followed by recovery of H4K16Ac to pre-treatment levels (Hsiao et al. 2013; Miller et al. 2010). In our work, H4K16Ac levels were assessed by immunofluorescence over different culture passages 1 h after Bleocin treatment (Fig. 2C and D). In comparison to control conditions (Fig. 1C and E), H4K16Ac levels slightly decreased in EP cells after 1 h of Bleocin treatment, however, these levels increased in LP cells, which showed similar levels in all passages tested (Fig. 2D). The achievement of a steady state level of H4K16Ac in all passages also coincided with convergence of 53BP1-γH2AX colocalization ratio values in all HDF passages (Fig. 2D).

Altogether, our data suggest that MOF-mediated H4K16 acetylation in response to DSB induction is influenced by in vitro aging. Thus, in response to DNA damage, H4K16 is deacetylated in EP cells to reach a specific level that most probably allows proper recruitment of 53BP1 to newly generated DSBs. In contrast, in vitro aged cells start from an already low H4K16Ac level that requires an upward adjustment after being threatened in order to achieve proper 53BP1 recruitment.

### Increased 53BP1 recruitment after DSB induction in aged HDFs depends on a specific H4K16 acetylation level

**Fig. 3:**
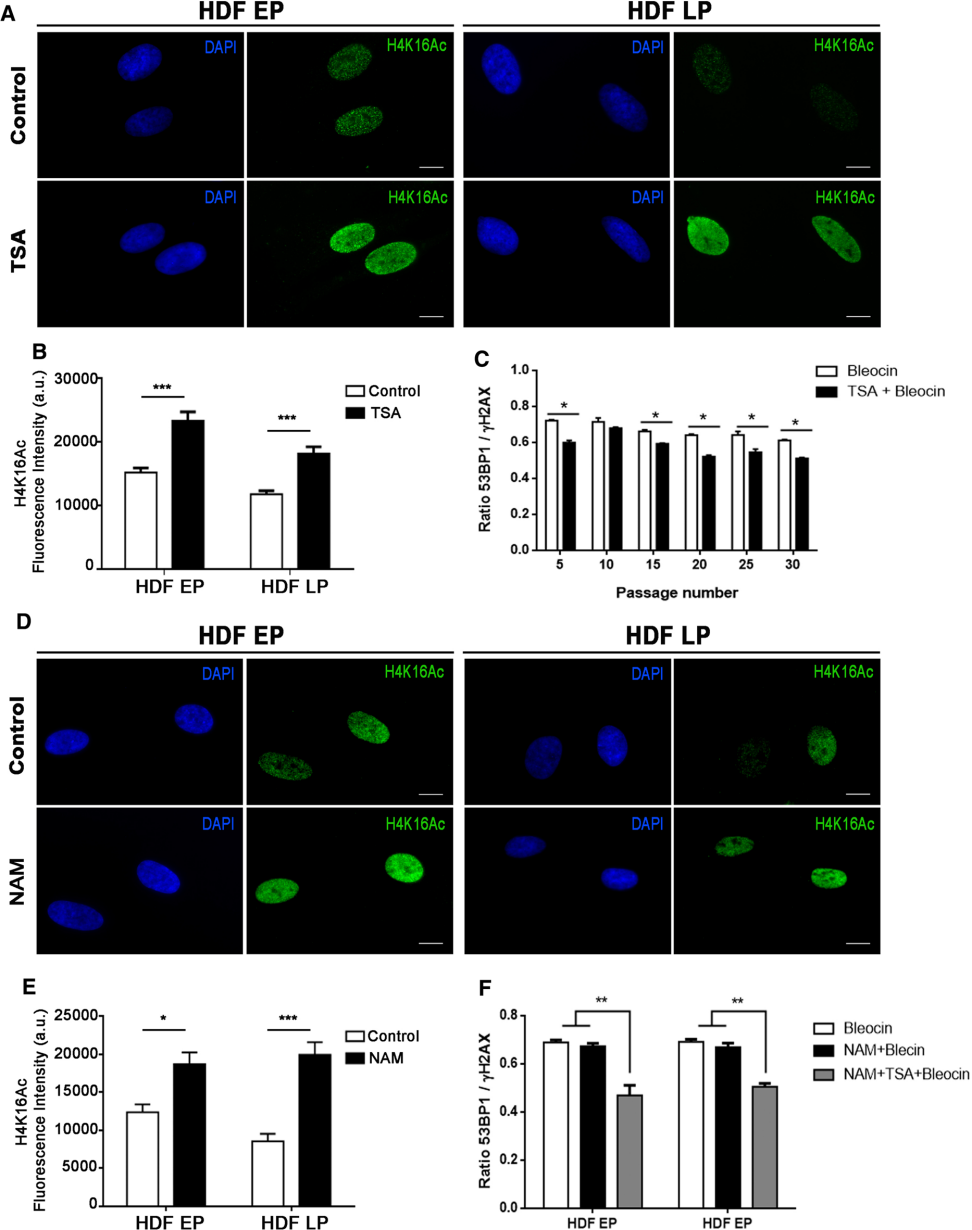
H4K16 hyperacetylation does not increase 53BP1 recruitment following DSB induction. **A** Immunostaining of cell nuclei with H4K16Ac (A488, green) in EP and LP HDFs under control conditions and after TSA treatment. Scale bar = 10 µm. **B** Mean of corrected total cell fluorescence intensity of H4K16Ac in control and TSA-treated EP and LP HDFs. Error bars indicate SEM and p-values indicate statistically significant differences (Mann–Whitney test; ***p < 0.001). **C** Mean of the ratios of 53BP1 and γH2AX foci colocalization in Bleocin and TSA + Bleocin-treated HDFs at various culture passages. Bar graph shows mean and SEM. p-values indicate the Student’s t-test significance levels; *p < 0.05. **D** Immunostaining of cell nuclei with H4K16Ac (A488, green) in EP and LP HDFs in control conditions and after NAM treatment. Scale bar = 10 µm. **E** Mean of corrected total cell fluorescence intensity of H4K16Ac in control and NAM-treated EP and LP HDFs. Error bars indicate SEM and p-values indicate statistically significant differences (Mann–Whitney test; *p < 0.05; ***p < 0.001) **F** Mean of the ratios of 53BP1 and γH2AX foci colocalization in EP and LP HDFs treated with Bleocin, NAM + Bleocin and NAM + TSA + Bleocin. Error bars indicate SEM and statistically significant differences are indicated by different letters (One-way ANOVA with Tukey test for multiple comparison; **p < 0.01). All the experiments were done in triplicates with -100–150 cells per experiment. DAPI (blue) was used to stain cell nuclei. a.u.: arbitrary units; EP: early passage (culture passage < 10); LP: late passage (culture passage > 20)

After DNA damage induction, aged HDFs displayed increases in both 53BP1-γH2AX colocalization and H4K16Ac levels. We reasoned that, if there was an association between both phenomena, then hyperacetylation could improve 53BP1 recruitment to DSBs. To elucidate this question, we analyzed 53BP1-γH2AX colocalization after induction of DSBs in HDFs treated with trichostatin A (TSA), which is an inhibitor of class I, II and IV HDACs (Kim et al. 2011). We observed that H4K16 acetylation levels were significantly higher in HDFs after TSA treatment (Fig. 3A and B). Contrary to our expectations, the colocalization ratio of 53BP1-γH2AX foci was reduced in all culture passages of TSA + Bleocin-treated cells in comparison to HDFs only treated with Bleocin (Fig. 3C, Table S3 and S4).

Since TSA is a general inhibitor of HDACs, we next checked the effect of nicotinamide (NAM), which is a specific inhibitor of sirtuins (Avalos et al. 2005), i.e., the major HDACs involved in H4K16Ac deacetylation (Vaquero et al. 2007) and whose expression remains unchanged in our in vitro aged cells. Again, the levels of H4K16Ac were significantly higher in NAM-treated HDFs in comparison to control HDFs (Fig. 3D and E); however, there were no differences in the 53BP1-γH2AX colocalization ratio between Bleocin-treated and NAM + Bleocin-treated cells in EP nor LP HDFs (Fig. 3F). Finally, when cells were treated with Bleocin and both hyperacetylators (NAM and TSA), the 53BP1-γH2AX ratio dropped in both EP and in LP cells (Fig. 3F). These results show that hyperacetylation does not translate into 53BP1-γH2AX ratio improvement in either case.

Considering all these results, we hypothesize that H4K16Ac has to reach an optimum level after DNA damage induction, which is critical for proper recruitment of 53BP1 to γH2AX-signalled DSBs. Thus, hyperacetylation beyond this level may not further improve, and possibly even hinders, the proper response to DNA damage.

### MOF silencing alters 53BP1 recruitment to induced DSBs

**Fig. 4:**
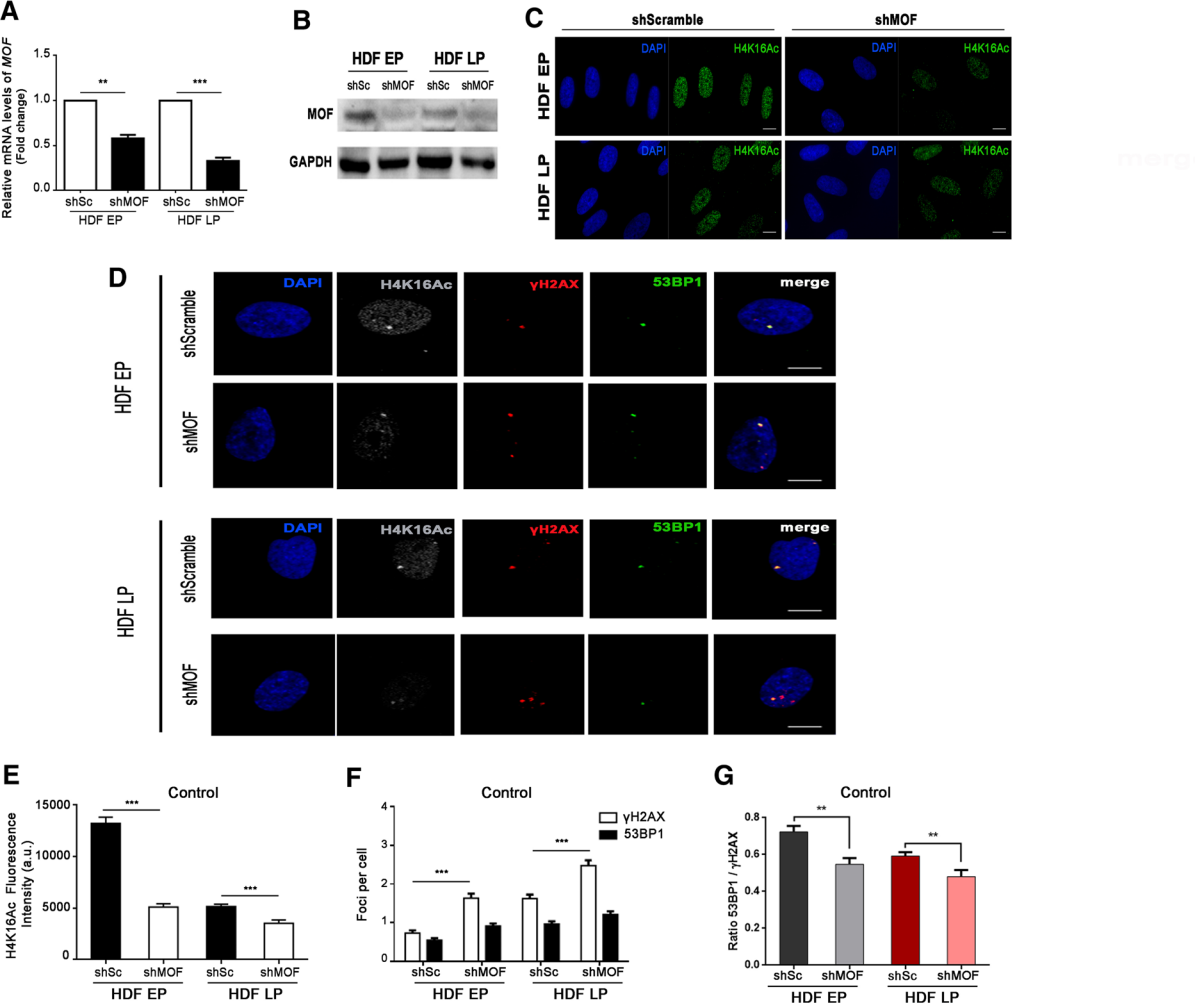
MOF depletion results in increased DSB induction in early and late passage HDFs. **A** Relative expression levels of MOF in EP and LP HDFs infected with shScramble (shSc) and shMOF. Data are presented as fold change with respect to shSc cells. The bar graph shows mean and SEM. p-values indicate the Student’s t-test significance levels; **p < 0.01, ***p < 0.001. **B** Western blot of MOF in EP and LP HDFs infected with shSc and shMOF. GAPDH was used as a loading control. **C** Immunostaining of cell nuclei with H4K16Ac (A488, green) in EP and LP HDFs infected with shSc and shMOF. **D** Triple immunofluorescence staining of H4K16Ac (A532, grey), γH2AX (A594, red) and 53BP1 (A488, green) in EP and LP HDFs infected with shSc and shMOF in control conditions. Merged panel shows γH2AX foci colocalizing with 53BP1 foci. **E** Mean of corrected total cell fluorescence intensity of H4K16Ac in EP and LP HDFs infected with shSc and shMOF. Error bars indicate SEM and p-values indicate statistically significant differences (Mann–Whitney test; ***p < 0.001). **F** Mean number of γH2AX and 53BP1 foci per cell in EP and LP HDFs infected with shSc and shMOF in control conditions. Error bars indicate SEM and p-values indicate statistically significant differences (Mann–Whitney test; ***p < 0.001). **G** Mean of the ratios of 53BP1 and γH2AX foci colocalization in EP and LP HDFs infected with shSc and shMOF in control conditions. Error bars indicate SEM and p-values indicate statistically significant differences (Mann–Whitney test; **p < 0.01). All the experiments were done in triplicates with 100–150 cells. Scale bar = 10 µm. DAPI (blue) was used to label and identify cell nuclei. a.u.: arbitrary units; EP: early passage (culture passage < 10); LP: late passage (culture passage > 20)

To further confirm our hypothesis on the steady state H4K16Ac level necessary for proper DNA damage response, we next investigated whether H4K16 hypoacetylation could impair 53BP1 recruitment to γH2AX-labelled DSBs. Given that MOF has been described to play a major role in H4K16 acetylation and our previous results confirm a relation between H4K16 acetylation in HDFs and MOF expression (Fig. 1G), we used a lentiviral vector expressing a short hairpin RNA (shRNA) to silence endogenous MOF (shMOF). Using RT-qPCR, we confirmed that EP and LP HDFs infected with shMOF showed down-regulation of MOF expression levels compared to cells infected with the control shRNA scramble (shScramble) (Fig. 4A). Additionally, Western blotting showed that MOF was reduced in HDFs after MOF depletion in comparison to the scramble control (Fig. 4B). As expected, MOF-depletion resulted in global H4K16 deacetylation, as assessed after immunostaining in EP and LP HDFs transduced with shMOF (Fig. 4C and D). Interestingly, after MOF silencing, EP cells showed H4K16Ac levels identical to those displayed by in vitro aged cells transduced with shScramble. MOF silencing in in vitro LP cells also translated into significantly decreased H4K16Ac levels, although this reduction was not as strong as in EP MOF-depleted cells (Fig. 4E).

We then examined the effect of MOF silencing and the subsequent hypoacetylation of H4K16 on 53BP1 recruitment. An increased frequency of γH2AX foci was observed in both EP and LP MOF-depleted HDFs (Fig. 4F). Although more DSBs were induced after MOF depletion, 53BP1 recruitment to those DSBs was not subsequently increased (Fig. 4F). Consequently, the 53BP1-γH2AX colocalization ratio decreased in MOF-depleted EP and LP HDFs (Fig. 4G), indicating that reduced H4K16Ac levels after MOF depletion favor DSB induction and hinder 53BP1 recruitment to these DSBs.

**Fig. 5:**
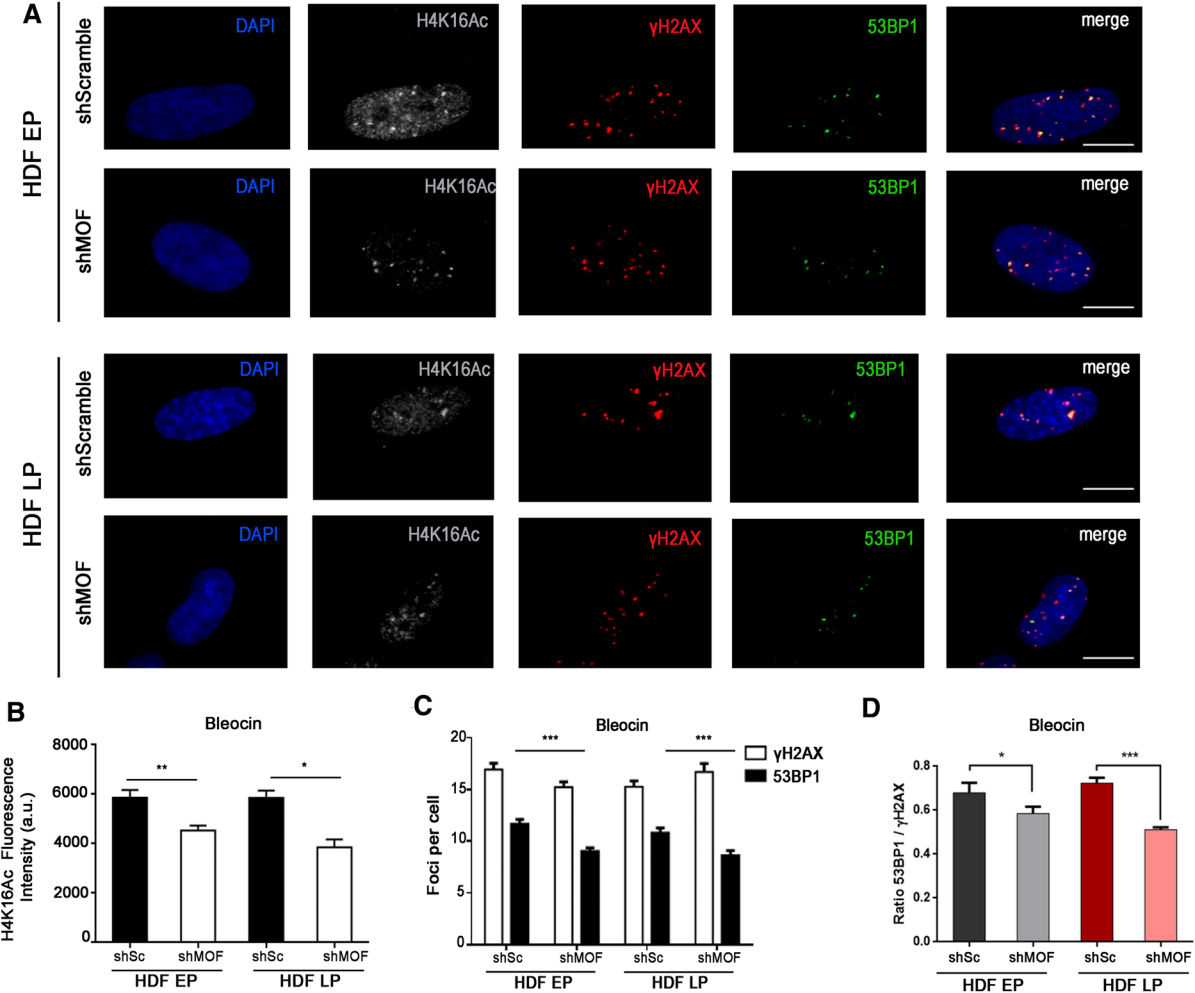
53BP1 recruitment is reduced in MOF-depleted HDFs after DNA damage induction. **A** Triple immunostaining of H4K16Ac (A532, grey), γH2AX (A594, red) and 53BP1 (A488, green) in EP and LP HDFs infected with shScramble (shSc) and shMOF after Bleocin treatment. The merge panel shows γH2AX foci colocalizing with 53BP1 foci. Cell nuclei were stained with DAPI (blue). Scale bar = 10 µm. **B** Mean of corrected total cell fluorescence intensity of H4K16Ac in EP and LP HDFs infected with shSc and shMOF after Bleocin treatment. Error bars indicate SEM and p-values indicate statistically significant differences (Mann–Whitney test; *p < 0.05, **p < 0.01;). **C** Mean number of γH2AX and 53BP1 foci per cell in EP and LP HDFs infected with shSc and shMOF after Bleocin treatment. Error bars indicate SEM and p-values indicate statistically significant differences (Mann–Whitney test; ***p < 0.001). **D** Mean of the ratios of 53BP1 and γH2AX foci colocalization in EP and LP HDFs infected with shSc and shMOF after Bleocin treatment. Error bars indicate SEM and p-values indicate statistically significant differences (Mann–Whitney test; *p > 0.05, ***p < 0.001). All the experiments were done in triplicates with 100–150 cells per experiment. a.u.: arbitrary units; EP: early passage (culture passage < 10); LP: late passage (culture passage > 20)

To further evaluate the effect of MOF depletion on 53BP1 recruitment, we analyzed the 53BP1-γH2AX colocalization ratio after inducing DSBs with Bleocin (Fig. 5A). MOF depletion was confirmed as reduced levels of H4K16Ac were found in cells infected with shMOF (Fig. 5B). After 1 h of treatment with Bleocin, the number of induced γH2AX foci increased in both control and MOF-depleted EP and LP HDFs, thus masking the MOF depletion-associated increase in γH2AX foci detected in untreated conditions (Fig. 5C). 53BP1 recruitment to Bleocin-induced γH2AX foci was significantly decreased in EP and LP HDFs after MOF inhibition (Fig. 5C), resulting in lower 53BP1-γH2AX colocalization ratios, especially in LP HDFs (Figs. 5D). Altogether these results indicate that H4K16Ac diminution after MOF depletion leads to increased γH2AX foci frequency and to a reduced recruitment of 53BP1 to those γH2AX foci.

## Discussion

In this work, we show that, as HDFs age, their H4K16Ac levels are lower with every passage when compared to younger cells. This observation correlates with increased γH2AX foci, a surrogate marker of unresolved DNA breaks. In a recent work, mouse cells from aged basal epidermis also exhibited markedly reduced levels of H4K16Ac (Dube et al. 2022). Loss of H4K16Ac is also characteristic of senescent cells in which it correlates with DNA compaction (Contrepois et al. 2012). Senescent cells also accumulate unrepaired DNA damage and show reduced repair efficiency (Campisi et al. 2013). In this work we have not used senescent cells, but cells aged in vitro. The results show that decreased H4K16 acetylation and increased unrepaired DNA damage are shared characteristics between senescent and in vitro aged cells, suggesting that these features become more pronounced with cell aging and may contribute to the eventual entry of cells into replicative senescence.

It has been described that, following DNA damage induction, H4K16 acetylation occurs in a biphasic pattern, with an initial wave of deacetylation followed by recovery of H4K16Ac to pre-treatment levels that may contribute to proper 53BP1 recruitment to DSBs (Hsiao et al. 2013; Miller et al. 2010). Nevertheless, the relationship between changes in H4K16Ac and 53BP1 recruitment has not been previously analyzed during in vitro aging. Our results confirm that, after DSB induction with Bleocin, a quick deacetylation occurs in EP HDFs, while in LP HDFs the overall lower levels of H4K16Ac increase. As a result, H4K16Ac levels reach a similar value in EP and LP HDFs and 53BP1-γH2AX colocalization ratios also converge in all passages. These results demonstrate that the response of H4K16 acetylation to DNA damage is modulated by age and that the biphasic pattern is not present in LP, in vitro aged cells. EP cells depart from high levels of H4K16Ac and respond by lowering them. Cells aged in vitro depart from low levels of H4K16Ac and respond by increasing them. In this way, both young and aged cells can reach the adequate level of H4K16Ac that allows proper recruitment of 53BP1 to DNA damage. Thus, after DNA insult, in vitro aged cells can overcome, at some extent, low H4K16Ac and the correlating DNA compaction by increasing H4K16Ac levels. Similar to this observation, higher levels of H4K16Ac facilitate 53BP1 recruitment after DSB induction in cellular models of premature aging, such as Zmpste24 − / − cells (Krishnan et al. 2011).

These results prompted us to speculate that a relationship may exist between H4K16Ac levels and 53BP1 recruitment efficiency to DSBs. Previous works have described an age-associated impairment in 53BP1 recruitment to DSBs, although the exact nature of this impairment remains to be described (Anglada et al. 2020). Given that H4K16Ac hyperacetylation leads to a more accessible and open chromatin state, elevating H4K16Ac levels using NAM and TSA could stimulate proper 53BP1 recruitment in in vitro aged cells. However, hyperacetylation failed to ameliorate the 53BP1-γH2AX ratio in both EP and LP HDFs. Similar to these findings, other studies have also shown that TSA strongly reduces 53BP1 foci formation after ionizing radiation (Fukuda et al. 2015), probably due to masking of the H4K20me2 binding motif of 53BP1 (Drané et al. 2017). Nonetheless, the results of this work clearly show that hyperacetylation per se*,* using either TSA or NAM, does not ameliorate 53BP1 recruitment in in vitro aged cells, and again, indicate that a specific H4K16Ac level must be reached in order to ensure proper 53BP1 recruitment to DSBs.

**Fig. 6:**
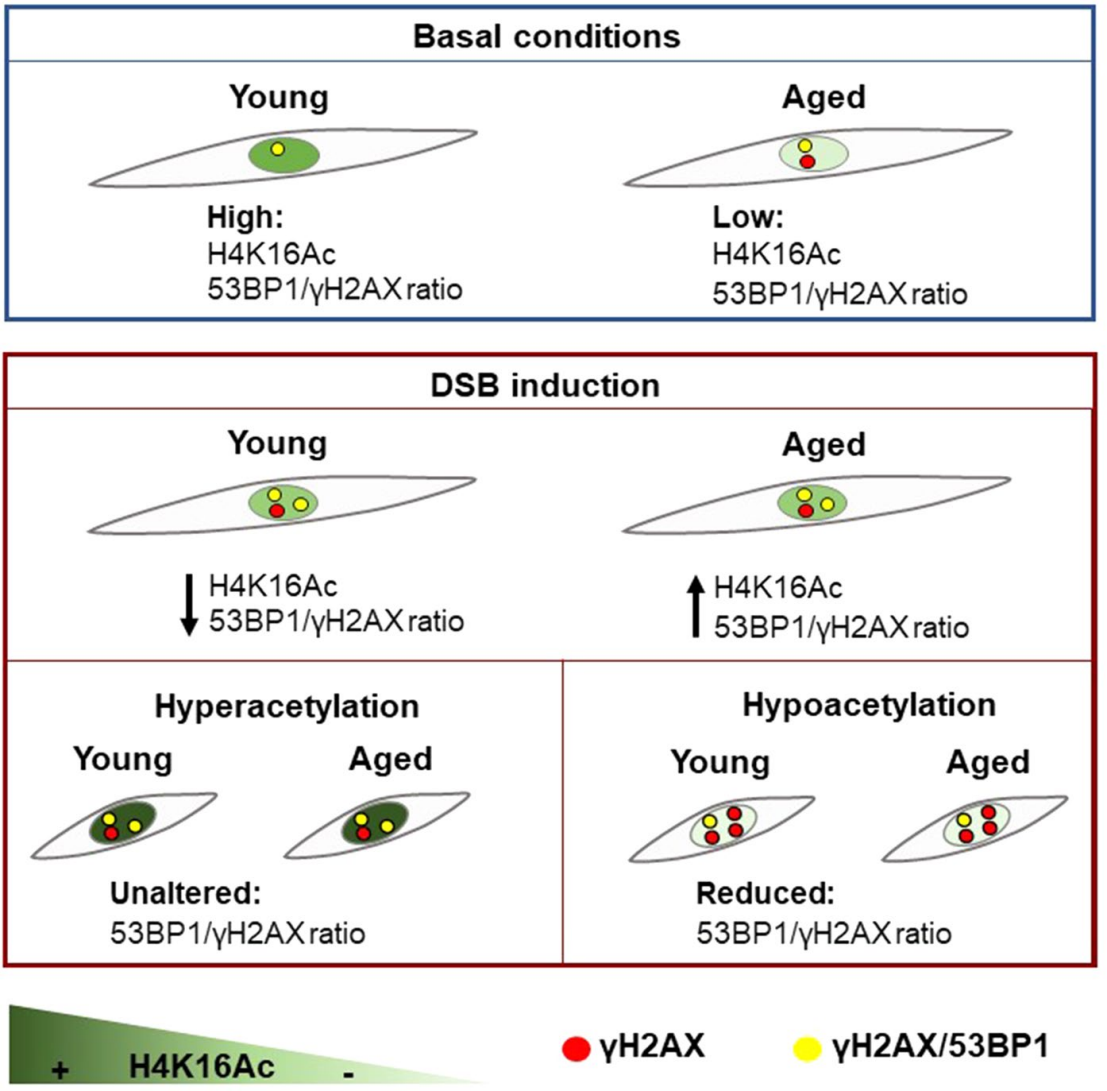
Model for the age-associated dynamics of H4K16Ac after DSB induction. Under basal conditions, the levels of H4K16Ac and the γH2AX-53BP1 colocalization ratio are higher in young, as opposed to aged, HDFs. After DSB induction, H4K16Ac levels and the 53BP1-γH2AX colocalization ratio slightly decrease in young cells, while these levels slightly increase in aged cells, smoothing the differences existing among culture passages in basal conditions. Independent from the culture passage, hyperacetylation prior to DSB induction does not improve the 53BP1-γH2AX colocalization ratio. In contrast, H4K16 hypoacetylation impairs 53BP1 recruitment to γH2AX-labelled DSBs, reducing the 53BP1-γH2AX colocalization ratio. Consequently, after DSB induction, H4K16Ac reaches a specific level required for appropriate recruitment of 53BP1 to DSB sites

To further study this hypothesis, we induced a global reduction of H4K16 acetylation that would prevent young and aged cells to reach the specific optimum H4K16Ac levels needed for proper DNA repair, and thus we expected to observe a decrease in 53BP1 recruitment in EP and LP HDFs. Hypoacetylation was achieved using shRNAs to deplete MOF expression. Previous studies have described sirtuins as important H4K16Ac deacetylases (Vaquero et al. 2007), but our results indicate that, during in vitro aging, sirtuin expression levels remain unchanged in our cell model, while MOF expression was clearly decreased. Indeed, MOF is the major H4K16 lysine acetyltransferase in Drosophila and mammals (Taipale et al. 2005; Smith et al. 2005). As expected, MOF depletion induced hypoacetylation in both young and aged cells. It also resulted in increased DNA damage and reduced 53BP1 recruitment to DSBs in EP and LP HDFs, and this defect was more pronounced after DSB induction. MOF silencing was more evident in LP than in EP HDFs, probably because the initial levels of MOF and H4K16Ac were already lower in LP HDFs compared to EP HDFs, reinforcing our results regarding the age-associated reduction of MOF and H4K16Ac. To our knowledge, the relation between MOF expression and in vitro aging has not been previously described. Previous studies have described that MOF depletion specifically affects the binding of repair mediator protein MDC1 to γH2AX, as well as the recruitment of other downstream repair proteins, such as 53BP1 and BRCA1, not by changing DDR protein levels, but by affecting the DDR through alterations at the chromatin level (Li et al. 2010; Sharma et al. 2010). Consistent with this observation, MOF overexpression in a mouse model of premature aging leads to enhanced global H4K16 acetylation levels and improved 53BP1 recruitment to DSB sites (Krishnan et al. 2011). Thus, the data presented in this study show that MOF and H4K16Ac levels are reduced during in vitro aging of HDFs and most probably contribute to the repair defect displayed by aged cells, as in vitro aged cells also present higher levels of γH2AX foci while recruitment of 53BP1 to these DSBs is impaired. Following DNA damage induction in young cells, H4K16Ac levels are reduced in order to reach a specific and optimum level required for an appropriate recruitment of 53BP1 to DSB sites. Meanwhile, aged cells increase their basal levels, reaching similar values of H4K16Ac to those shown in young HDFs. Thus, MOF-dependent H4K16Ac appears as another age-dependent marker that directly impacts the DNA repair ability of aged cells (Fig. 6). Future models should include the use of other cell lines besides HDFs to verify and shed more light on the relationship between age-related H4K16Ac changes and age-associated DSBs repair capacity.

## Conclusions

Our study demonstrates that the MOF-dependent reduction in H4K16Ac levels during in vitro aging in human fibroblasts contributes to the repair defect displayed by in vitro aged cells and may eventually compromise any proper response to DNA damage, most probably leading the cell to senescence. Age-mediated variations in H4K16Ac following the activation of the DNA damage response point at this histone mark as a key mediator between DNA repair and age-associated chromatin alterations. These results highlight the importance of reviewing already described cellular responses during aging, since it can lead to changes in diagnostic markers or therapeutic targets. Future efforts addressing the exact mechanism by which MOF-dependent H4K16 deacetylation impairs 53BP1 recruitment to DSBs could be the key to restore DNA repair fidelity during aging, prevent a rise in genomic instability in older individuals and delay senescence entry.

## Supplementary Information

Below is the link to the electronic supplementary material.

Supplementary file1 (PDF 512 KB) Flow cytometry analysis for cell cycle distribution of early and late passage HDFs

Supplementary file2 (PDF 554 KB) HDAC1, HDAC2 and TIP60 mRNA expression levels are not affected in in vitro aging

Supplementary file3 (PDF 12 KB) Primers sequences of genes tested by RT-qPCR. F: forward; R: reverse

Supplementary file4 (PDF 10 KB) Mean number of γH2AX and 53BP1 foci per cell in HDFs at different culture passages in control conditions

Supplementary file5 (PDF 21 KB) Mean number of γH2AX and 53BP1 foci per cell in HDFs at different culture passages after bleocin treatment

Supplementary file6 (PDF 21 KB) Mean number of γH2AX and 53BP1 foci per cell in HDFs at different culture passages after TSA+bleocin treatment

## Data Availability

The datasets used and/or analyzed during the current study are available from the corresponding author on reasonable request.
