# Supplementary figures and images for: Role of H4K16 acetylation in 53BP1 recruitment to double-strand break sites in in vitro aged cells
Source: Biogerontology. 2022 Jul 18;23(4):499–514. doi: 10.1007/s10522-022-09979-6 (PMC9388460; doi:10.1007/s10522-022-09979-6)

# HDF EP

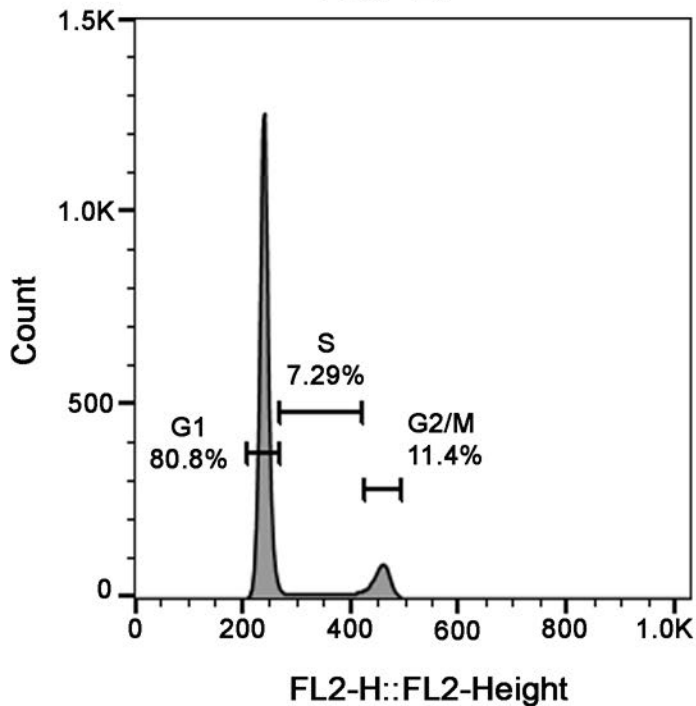

# HDF LP

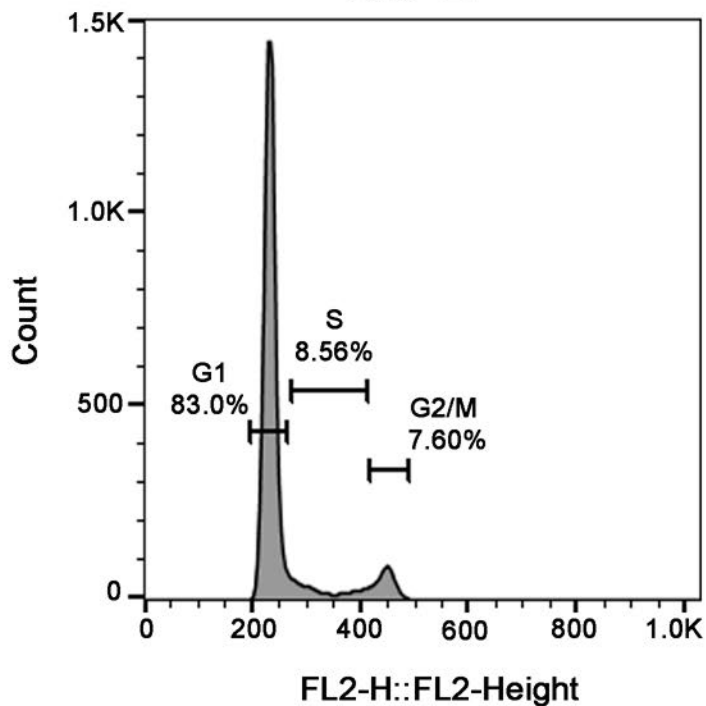

Supplement: Supplementary file 1 — Supplementary file1 (PDF 512 KB) Flow cytometry analysis for cell cycle distribution of early and late passage HDFs [file 10522_2022_9979_MOESM1_ESM.pdf]

### ***HDAC1***

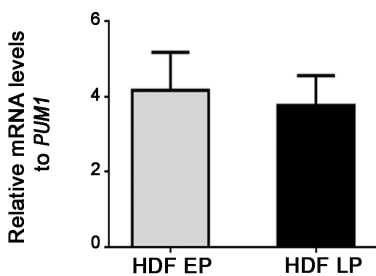

### ***HDAC2***

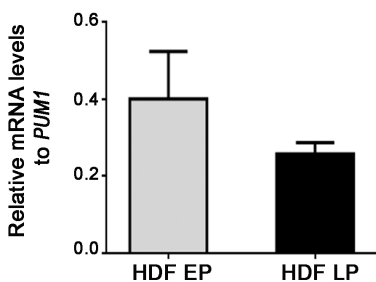

### ***TIP60***

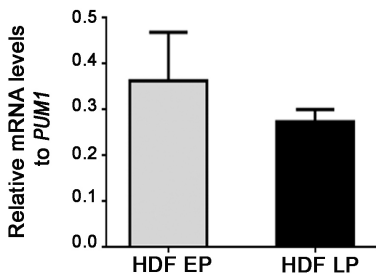

Supplement: Supplementary file 2 — Supplementary file2 (PDF 554 KB) HDAC1, HDAC2 and TIP60 mRNA expression levels are not affected in in vitro aging [file 10522_2022_9979_MOESM2_ESM.pdf]
