# Supplementary material for: Role of H4K16 acetylation in 53BP1 recruitment to double-strand break sites in in vitro aged cells
Source: Biogerontology. 2022 Jul 18;23(4):499–514. doi: 10.1007/s10522-022-09979-6 (PMC9388460; doi:10.1007/s10522-022-09979-6)
Supplement: Supplementary file 3 — Supplementary file3 (PDF 12 KB) Primers sequences of genes tested by RT-qPCR. F: forward; R: reverse [file 10522_2022_9979_MOESM3_ESM.pdf]

MOF	F: GAAGTCACGGTGGAGATCGG	
	R: AATTCCTCTCGGCCCTCCT	
SIRT1	F: TGACTGTGAAGCTGTACGAGG	
	R: TGGTTTCATGATAGCAAGCGG	
SIRT2	F: GCTGAAGGACAAGGGGCTAC	
	R: CCACCAAGTCCTCCTGTTCC	
HDAC1	F: GGACCAGATTTCAAGCTCCA	
	R: CGGCAGCATTCTAAGGTTCT	
HDAC2	F: TCCTCCAGCCCAATTAACAG	
	R: AGCTCTCAACTGGCGGTTC	
TIP60	F: TGCGAGTTCTGCCTCAAGTA	
	R: ATCTCATTGCCTGGAGGATG	
PUM1	F: CGGTCGTCCTGAGGATAAAA	
	R: CGTACGTGAGGCGTGAGTAA	

F: forward; R: reverse;
