# Supplementary material for: Role of H4K16 acetylation in 53BP1 recruitment to double-strand break sites in in vitro aged cells
Source: Biogerontology. 2022 Jul 18;23(4):499–514. doi: 10.1007/s10522-022-09979-6 (PMC9388460; doi:10.1007/s10522-022-09979-6)
Supplement: Supplementary file 4 — Supplementary file4 (PDF 10 KB) Mean number of γH2AX and 53BP1 foci per cell in HDFs at different culture passages in control conditions [file 10522_2022_9979_MOESM4_ESM.pdf]

**Culture Passage**	**γH2AX**	**St. Error**	**53BP1**	**St.Error**
P5	0,64	0,08	0,60	0,071
P10	1,04	0,10	0,81	0,08
P15	1,23	0,14	0,81	0,08
P20	2,07	0,15	1,04	0,07
P25	2,45	0,20	1,04	0,07
P30	3,88	0,25	1,73	0,12
