# Supplementary material for: Role of H4K16 acetylation in 53BP1 recruitment to double-strand break sites in in vitro aged cells
Source: Biogerontology. 2022 Jul 18;23(4):499–514. doi: 10.1007/s10522-022-09979-6 (PMC9388460; doi:10.1007/s10522-022-09979-6)
Supplement: Supplementary file 5 — Supplementary file5 (PDF 21 KB) Mean number of γH2AX and 53BP1 foci per cell in HDFs at different culture passages after bleocin treatment [file 10522_2022_9979_MOESM5_ESM.pdf]

Culture Passage	γH2AX	St. Error	53BP1	St.Error
P5	8,55	0,39	6,18	0,39
P10	9,26	0,49	6,79	0,36
P15	8,29	0,43	5,61	0,31
P20	12,38	0,48	8,06	0,368
P25	12,48	7,93	7,93	0,34
P30	11,95	0,47	7,22	0,32
